# Multifaceted elucidation of aminoguanidine in protecting against diabetes-induced vascular endothelial injury: Aminoguanidine protects against diabetic endothelial injury

**DOI:** 10.3724/abbs.2026005

**Published:** 2026-02-03

**Authors:** Huiru Tang, Yuhan Zhai, Gaojun Wang, Junfeng Shi, Yuxue Li, Zhipeng Wang, Yudong Guan, Kexin Zhang, Wenshuang Wang, Qinying Li, Xiaodong Sun, Hongyan Qiu

**Affiliations:** 1 Department of Endocrinology and Metabolism Affiliated Hospital of Shandong Second Medical University Weifang 261031 China; 2 Clinical Research Center Affiliated Hospital of Shandong Second Medical University Weifang 261031 China; 3 School of Laboratory Medicine Shandong Second Medical University Weifang 261031 China; 4 Weifang Maternal and Child Health Hospital Weifang 261031 China; 5 Oral and Maxillofacial Surgery Department Affiliated Hospital of Shandong Second Medical University Weifang 261031 China; 6 Department of Thoracic Surgery Affiliated Hospital of Shandong Second Medical University Weifang 261031 China; 7 School of Stomatology Henan University Kaifeng 475001 China; 8 Zhongzhou Laboratory Zhengzhou 450046 China; 9 National Glycoengineering Research Center Shandong Key Laboratory of Carbohydrate Chemistry and Glycobiology and State Key Laboratory of Microbial Technology and Carbohydrate-conjugate Drugs Shandong University Qingdao 266237 China

**Keywords:** diabetes, vascular endothelial injury, aminoguanidine, glycocalyx, metabolomics

## Abstract

Chronic hyperglycemia-driven protein glycation in diabetes is a key pathogenic factor in vascular endothelial injury. This study demonstrates the multifaceted protective profile of aminoguanidine (AMG) against diabetes-induced vascular injury. As a carbonyl scavenger, AMG effectively traps methylglyoxal (MGO), inhibiting advanced glycation end products (AGEs) formation while preserving endothelial glycocalyx integrity and permeability. Mechanistically, AMG suppresses NF-κB-mediated inflammation, upregulates the eNOS/NO pathway, and restores CD31 expression, collectively mitigating oxidative stress, apoptosis and impaired proliferation in human umbilical vein endothelial cells (HUVECs). Metabolomic profiling further reveals AMG’s capacity to alleviate MGO-induced metabolic dysregulation by modulating critical pathways, including glutathione metabolism and the TCA cycle. In diabetic mice, AMG attenuates site-specific glycation adducts on plasma albumin and demonstrates significant therapeutic efficacy by improving endothelial-dependent vasodilation via the eNOS/NO pathway, reducing vascular fibrosis and basement membrane thickening, and suppressing NF-κB-driven inflammatory responses. These integrated findings establish AMG as a promising therapeutic candidate with multifaceted protective effects against diabetic vascular injury.

## Introduction

Diabetes is a chronic disease characterized by hyperglycemia [1]. Chronic hyperglycemia induces endothelial dysfunction, primarily through non-enzymatic glycation, a key pathogenic mechanism in diabetic vascular complications [2]. Non-enzymatic glycation is a nucleophilic addition reaction between glucose and basic amino acids of proteins [3]. This process generates highly reactive carbonyl intermediates, such as methylglyoxal (MGO), glyoxal and deoxyglucosone, which exhibit significantly greater reactivity than glucose molecules. These carbonyl compounds can rapidly react with plasma proteins to form toxic advanced glycation end products (AGEs), which accumulate in plasma and contribute to pathological effects [4].

As the predominant plasma protein, human serum albumin (HSA) undergoes substantial glycation under diabetic conditions. It constitutes over 80% of circulating glycated proteins and is the major source of AGEs in the circulation [ 5, 6] . AGEs bind to the receptor for advanced glycation end products (RAGE) on the surface of vascular endothelial cells, activating a series of signaling pathways, including nuclear factor kappa-B (NF-κB), which culminates in apoptosis, oxidative stress, chronic inflammation, and metabolic disorders in endothelial cells [7]. These interconnected pathological mechanisms synergistically compromise vascular integrity by disrupting endothelial barrier function, reducing nitric oxide (NO) bioavailability, and impairing endothelium-dependent vasodilation, thereby promoting the progression of diabetic vascular complications [8].

The endothelial glycocalyx, a dynamic meshwork of proteoglycans, glycoproteins, and adsorbed plasma proteins, serves as the primary vascular barrier [9]. It regulates the permeability of macromolecules across the endothelium and plays a pivotal role in endothelial protection, homeostasis, and hemodynamic regulation [10]. However, the precise structural and functional perturbations induced by diabetic conditions, particularly MGO-mediated glycocalyx damage, remain insufficiently characterized.

Aminoguanidine (AMG), a prototypical anti-glycation drug, exerts its therapeutic effects by competitively binding to reactive carbonyl compounds ( *e*. *g*., MGO) through its free amino groups, thereby inhibiting protein glycation, reducing AGEs accumulation, and ameliorating diabetic vascular complications [11]. Extensive research has established the vascular protective properties of AMG. These include the upregulation of large-conductance Ca ^2^^+^-activated K ^+^-β1 (BK-β1) protein expression to improve coronary function, attenuation of protein oxidation, and restoration of mitochondrial autophagic flux. Additionally, AMG mitigates oxidative stress via the protein kinase B/Forkhead box protein O1 (AKT/FOXO1) signaling pathway [ 12, 13] . Furthermore, AMG has been shown to effectively downregulate key pathological markers in aged kidneys, including fibrosis markers (p38/α-SMA, p38/α-smooth muscle actin) and CHOP (C/EBP homologous protein)), senescence markers (p53 and p21), and oxidative stress markers (4-hydroxynonenal) [14].

However, the current understanding of AMG’s actions remains superficial, with critical gaps in site-specific glycation inhibition on serum albumin, systemic metabolic profiling in endothelial cells, and glycocalyx preservation mechanisms against MGO insult [15]. By integrating multi-omics analyses (glycomics, metabolomics, and proteomics), this study systematically investigates AMG’s role, from deciphering its molecular function in glycation inhibition and mapping its endothelial metabolic network to elucidating glycocalyx restoration and the recovery of endothelial nitric oxide synthase/nitric oxide (eNOS/NO)-mediated vasodilation. These findings will advance the development of targeted therapies for diabetic endothelial dysfunction by revealing AMG’s multidimensional protective function, including preservation of glycocalyx integrity, site-specific inhibition of albumin glycation, and regulation of metabolic homeostasis.

## Materials and Methods

### Inhibition of non-enzymatic glycation of HSA by AMG *in vitro*

HSA (Sigma-Aldrich, St Louis, USA) was equally divided into 2 groups: the control group contained 35 mg/mL HSA and 100 mM glucose in PBS buffer, while the experimental group was supplemented with 33.3 mM AMG (Sigma-Aldrich). All samples were incubated at 37°C for 30 days. Fluorescence measurements of AGEs (Ex/Em = 355/460 nm) were performed every 48 h using a multifunctional full-wavelength microplate reader (SuPerMax 3100; Flash, Shanghai, China).

### Cell culture

Human umbilical vein endothelial cells (HUVECs; FH1199) were obtained from Shanghai Fuheng Biotechnology (Shanghai, China) and cultured in DMEM (Servicebio, Beijing, China) supplemented with 10% fetal bovine serum (FBS; VivaCell, Shanghai, China) and 1% penicillin-streptomycin (Servicebio) at 37°C. All experimental procedures were performed using cells between passages 5–20 at 80%–90% confluence.

### Cell physiology and function experiment

Cells were treated with normal culture medium (CT group), medium containing 800 μM MGO (Macklin, Shanghai, China) (MGO group), and medium containing 800 μM MGO plus 400 μg/mL AMG (AMG group). Cell viability was assessed using the CCK8 assay kit (Servicebio). An NO detection kit (Beyotime, Shanghai, China) was utilized to quantify NO release with or without L-NAME (100 μM, an eNOS inhibitor; Beyotime). Cell counts and morphological images were also obtained after 24 h.

Apoptosis and reactive oxygen species (ROS) assays were conducted using Annexin V-FITC/PI (Yeasen Biotechnology, Shanghai, China) and ROS (Beyotime) detection kits. Following probe incubation, the data were analyzed via flow cytometry (DxFLEX; Beckman Coulter, Pasadena, USA). Each experiment was performed in triplicate to ensure result reproducibility.

### Western blot analysis

Total protein was extracted from HUVECs using RIPA lysis buffer (Solarbio, Wuhan, China), followed by standard western blot procedures. Primary antibodies against CD31 (Uping Bio, Hangzhou, China), eNOS (endothelial nitric oxide synthase; Abcam, Cambridge, UK), NF-κB (Cell Signaling Technology, Danvers, USA), and GAPDH (Proteintech, Wuhan, China) were used for protein detection, while horseradish peroxidase (HRP)-conjugated goat anti-rabbit IgG (H + L) and goat anti-mouse IgG (H + L) (Beyotime) were employed as secondary antibodies. Blots were developed using ECL (YANXI, Shanghai, China) substrate for detection.

### Quantitative analysis of GAGs on the cell membrane surface and membrane permeability experiments

For labeling glycosaminoglycans (GAGs), 5 μg/mL supercharged green fluorescent protein (ScGFP, a gift from Dr Wenshuang Wang, National Glycoengineering Research Center, Shandong University, Jinan, China) was employed as a molecular probe. Following fixation of cells with 4% paraformaldehyde (Biosharp, Beijing, China), cell nuclei were stained with the blue fluorescent dye DAPI (Beyotime). Fluorescence imaging of the 3 groups of HUVECs was conducted using a live cell imaging system (EVOS ^TM^M7000; Thermo Fisher Scientific, Waltham, USA).

In the GAGs quantification experiment, over 5 × 10 ^6^ cells were collected and lysed. Equal amounts of protein were extracted from each group. Glycoproteins were precipitated overnight at –20°C using ice-cold acetone (1:3; v/v). After removing organic solvents, pronase protease (Sigma-Aldrich) was used to separate glycan chains from proteins. Chondroitinase ABC (Bicheng Biotechnology, Beijing, China) and heparinase I/II/III (BioKangTai, Shenzhen, China) were applied to cleave glycan chains into disaccharide units. Disaccharide units were labeled with 0.05 M 2-aminoacridone (2-AMAC; Sigma-Aldrich) and reduced with 0.5 M sodium cyanoborohydride (Sigma-Aldrich). Labeled disaccharides were analyzed by LC-MS/MS MRM (liquid chromatography-tandem mass spectrometry, multiple reaction monitoring) mode. Targeted quantification of disaccharide composition units was performed based on the content of 14 disaccharides in the GAGs standard.

The cell membrane permeability experiment was conducted using serum-free medium diluted with FITC-BSA (100 μg/mL; Solarbio). After incubation at 37°C for 1 h, the cells were fixed with 4% paraformaldehyde for 15 min, and green fluorescence images were captured using a live cell imaging system (Thermo Fisher Scientific).

### Cell metabolomics and ^13^C-labeled metabolic flux analysis

For metabolite extraction, 1 mL of precooled 80% methanol (Thermo Fisher Scientific) was added to 2 × 10 ^6^ cells. The supernatant was collected by centrifugation after sonication and then evaporated to dryness. The dried samples were re-suspended in 50% methanol and analyzed by LC-MS/MS using a QE Plus Mass spectrometer (Thermo Fisher Scientific). Solvent A was a 0.1% formic acid (FA; Thermo Fisher Scientific) aqueous solution, and solvent B was a 100% methanol solution. Metabolite detection was carried out in both positive and negative ion modes at a flow rate of 0.3 mL/min. The metabolites were searched using Compound Discoverer 3.0 software ( https://www.thermofisher.com/CompoundDiscoverer).

For metabolic flux analysis, HUVECs were starved overnight in carbon-free medium (glucose-free, glutamine-free, and serum-free; Servicebio). Subsequently, 5 mM ^1^ ^3^C-labeled glucose (Energy Chemical, Hefei, China) was added along with MGO or AMG for 24 h. A total of 1.5 × 10 ^6^ cells were extracted using pre-chilled 80% methanol and analyzed by LC-MS/MS.

Differentially abundant metabolites and related pathways were screened using R language (R-4.4.3) and the MetaboAnalyst (6.0) website [Kyoto Encyclopedia of Genes and Genomes (KEGG) modules], with criteria of fold change (FC) > 1.3 and *P*  < 0.05.

### Animal experiments

Eighteen 6-week-old male C57BL/6J SPF mice were purchased from Jinan Pengyue Laboratory Animal Breeding Company (Jinan, China). Six mice were randomly assigned to the control group, while the remaining 12 were switched to a 60 kcal% high-fat diet (Wuxi FanBo Biologics, Wuxi, China) for one month. The type 2 diabetes model (T2DM) was established by intraperitoneal injection of streptozotocin (STZ, 50 mg/kg; Sigma-Aldrich) for 4 consecutive days. Successful modeling was confirmed if random blood glucose levels exceeded 11.1 mM one week later. Six mice in the AMG group received intraperitoneal injections of 50 mg/kg AMG for 45 consecutive days. All experimental procedures were performed in strict accordance with the ARRIVE guidelines and were approved by the Shandong Second Medical University Ethics Committee (2024SDL751).

### Vascular vasoconstriction and vasodilation function measurement

C57 male mice were anesthetized with isoflurane gas, and the aorta was carefully extracted. The vessels were cut into 3 equal ring segments (2–4 mm each). The vascular rings were suspended in Krebs-Henseleit buffer under an initial tension of 1 g and equilibrated for 60 min to achieve a stable state. MGO (800 μM) was added to the MGO group, while 800 μM MGO and 400 μg/mL AMG were added to the AMG group. Endothelial-dependent vasodilation was pre-contracted with norepinephrine (NE, 10 ^-3^ M; Shanghai Yingxin Laboratory Equipment Co., Ltd., Shanghai, China) until the stable plateau phase, followed by cumulative addition of acetylcholine (ACh, 10 ^–8^–10 ^–4^ M; Sigma-Aldrich). Changes in vascular ring tension were recorded using an animal pressure volume measuring device (PanLab; Harvard Apparatus, Holliston, USA).

### Identification of non-enzymatic glycation sites in plasma albumin

Plasma samples were collected from mice. PEG-6000 (Bangon Biotech, Shanghai, China) was used to precipitate globulins, and the albumin-containing supernatant was separated. Albumin was denatured by SDC-urea buffer (8 M; Sigma-Aldrich). Equal amounts of 200 μg albumin were reduced by 8 mM DTT (dithiothreitol; Sigma-Aldrich) and alkylated with 16 mM IAA (iodoacetamide; Sigma-Aldrich). Peptides were generated by overnight trypsin digestion (1:25, w/w; Promega, Madison, USA) at 37°C. A 20 μg aliquot of the peptides was desalted, and then 100 ng was loaded on a C18 column (Resil-Pur, 3 μm, 75 μm × 250 mm) for NanoLC-MS/MS (Orbitrap Eclipse Mass Spectrometer; Thermo Fisher Scientific) analysis. Proteome Discoverer software was used to search for peptides. The relative peak areas of non-enzymatic glycation sites were used to compare the degree of glycation.

### Vascular section staining and immunohistochemistry

Mouse vessels were dissected and placed in 4% paraformaldehyde for fixation. Paraffin embedding and sectioning were performed at 4 μm for hematoxylin and eosin (H&E), Masson, and Sirius red staining. Immunohistochemical staining was performed using NF-κB primary antibody (Cell Signaling Technology) and corresponding secondary antibody. The sections were subsequently placed in 3,3′-diaminobenzidine chromogen solution and incubated at room temperature for 5–10 min until a brown reaction product appeared. Nuclei were counterstained with hematoxylin, and the sections were dehydrated and mounted. All these conditions of the vessels were observed under a live cell imaging system (EVOS ^TM^M7000; Thermo Fisher Scientific).

### Statistical analysis

Data were analyzed statistically using GraphPad Prism 9.5 (Boston, USA). Each group had at least 3 biological replicates. Depending on the data type, either one-way ANOVA of variance or unpaired Student’s *t* tests were selected. *P*  < 0.05 indicated a significant difference.

## Results

### AMG effectively inhibits the non-enzymatic glycation of HSA *in vitro*

**Figure 1:**
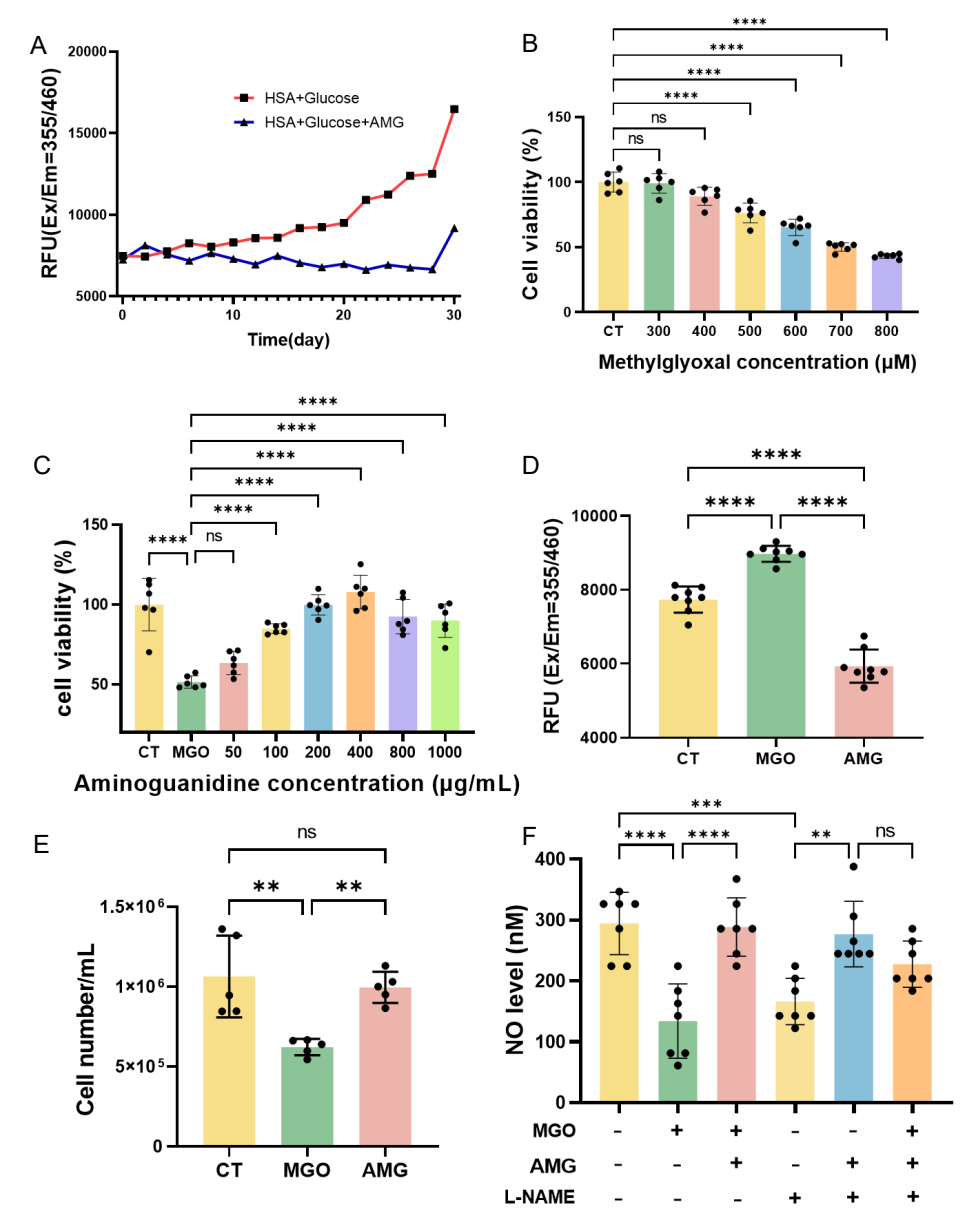
Figure 1 AMG inhibits AGEs formation and promotes NO production in HUVECs (A) AGEs generation during HSA, Glu, and AMG co-incubation. (B) Endothelial cell proliferation viability changes under 300–800 μM MGO (n = 6). (C) Proliferation viability changes with 50–1000 μg/mL AMG under 800 μM MGO stimulation (n = 6). (D) Inhibitory effect of 400 μg/mL AMG on AGEs generation in cell supernatant under 800 μM MGO (n = 8). (E) Cell number recovery with 400 μg/mL AMG under 800 μM MGO stimulation (n = 5). (F) NO content changes with 400 μg/mL AMG under 800 μM MGO and 100 μM L-NAME (n = 7). Data were analyzed by one-way ANOVA. **P < 0.01, ***P < 0.001, ****P < 0.0001. ns, not significant.

To investigate the inhibitory effect of AMG on plasma high-abundance HSA glycation, 30 mg/mL HSA and 100 mM glucose were co-incubated at 37°C for 30 days ( Figure 1A). The experimental group additionally contained 33.3 mM AMG (a 3:1 molar ratio of glucose to AMG was adopted based on their molecular structures). AGEs-specific characteristic fluorescence intensity was monitored every 48 h to evaluate the degree of non-enzymatic glycation. With prolonged incubation, the AMG group showed lower AGEs fluorescence than the glucose-only group, confirming AMG’s suppression of glycation.

### AMG attenuates the formation of AGEs induced by MGO in HUVECs

MGO, a non-enzymatic glycation intermediate with higher reactivity, was used to induce injury. To rapidly model endothelial cell injury, HUVECs were exposed to MGO at concentrations of 300–800 μM ( Figure 1B). Cell viability decreased progressively with increasing MGO concentration, reaching 43% of the control group at 800 μM ( *P*  < 0.0001). This concentration was selected for subsequent experiments. Then, the effect of AMG (50–1000 μg/mL) on restoring cell viability under MGO stimulation was assessed ( Figure 1C). The highest viability was achieved at 400 μg/mL ( *P*  < 0.0001), which was subsequently used for further experiments. Meanwhile AGEs fluorescence was significantly lower in the AMG group than in the MGO group ( *P*  < 0.0001; Figure 1D), indicating that AMG suppressed AGEs formation. Cell count changes mirrored the same viability trend ( Figure 1E).

### AMG restores eNOS/NO and reduces oxidative stress to protect endothelial cells

The eNOS/NO pathway is the primary route for endothelial NO release, and NO detection assays revealed that NO levels significantly decreased after MGO stimulation but rebounded with AMG treatment ( *P*  < 0.0001; Figure 1F). The eNOS inhibitor L-NAME (100 μM) was used to investigate the effects of AMG under different conditions. The results showed that L-NAME suppressed NO levels in HUVECs by inhibiting eNOS. This suppression was alleviated by subsequent AMG treatment. Moreover, AMG also attenuated eNOS suppression even under co-treatment with MGO and L-NAME. These findings demonstrate that AMG ameliorates endothelial injury by protecting the eNOS/NO pathway.

MGO-induced endothelial damage and AMG’s restorative effects were distinctly manifested in cell morphology ( Supplementary Figure S1). The cell morphology in the CT group exhibited a normal, spindle-shaped morphology, while MGO treatment caused rounding and increased cell death. However, the presence of AMG normalizes cell morphology and even more effectively delays natural cell aging and death.

**Figure 2:**
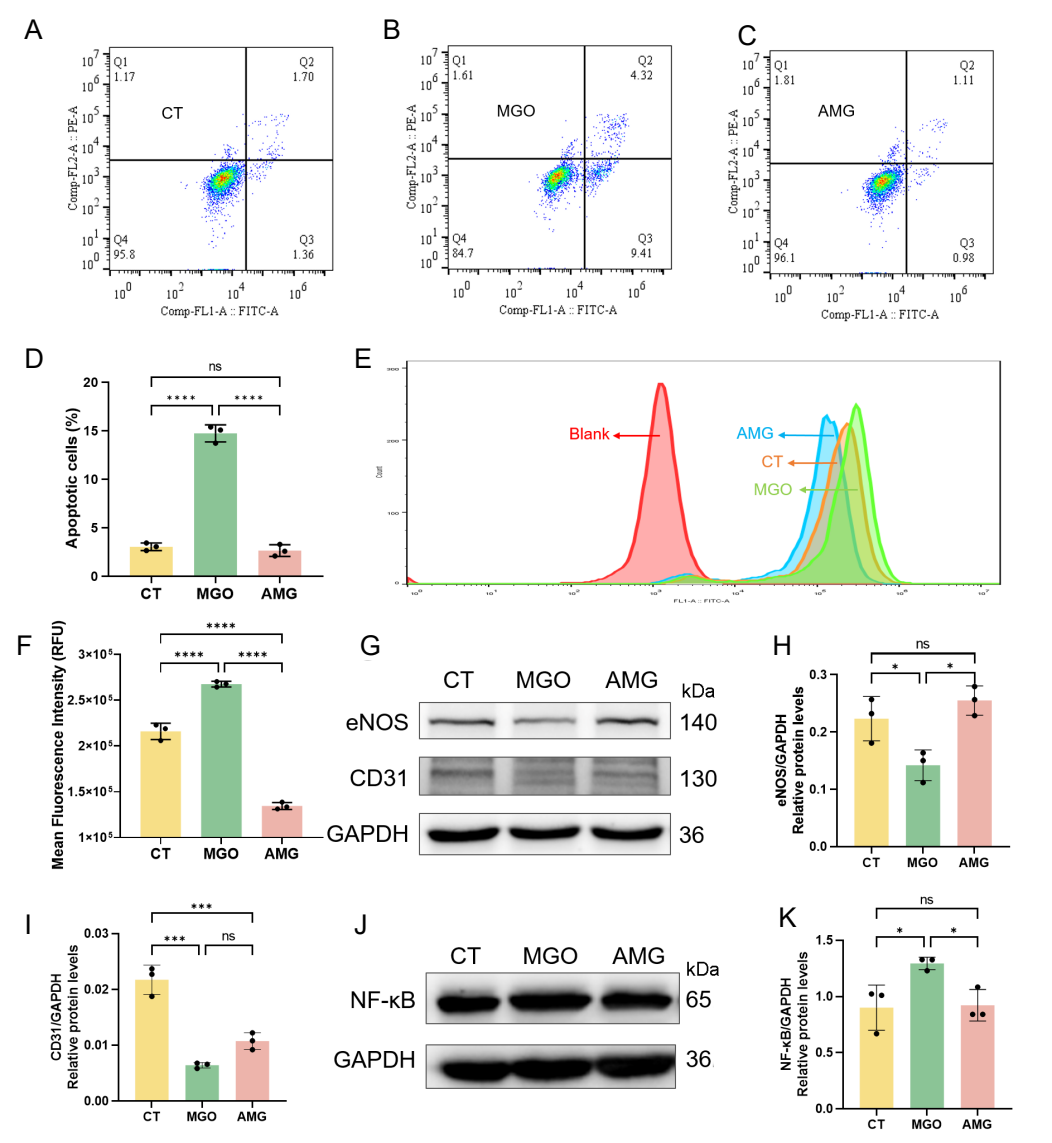
Figure 2 AMG attenuates MGO-induced endothelial injury by suppressing apoptosis, oxidative stress, and NF-κB and activating eNOS/CD31 (A–C) Recovery of apoptosis in MGO-stimulated cells with AMG intervention: A (CT), B (MGO), and C (AMG). (D) Statistical analysis of apoptosis rates in 3 groups (n = 3). (E,F) Comparison of oxidative stress levels in MGO-stimulated cells with AMG intervention (n = 3). (G–K) Western blot and statistical analysis of eNOS, CD31, and NF-κB protein expressions. Data were analyzed by one-way ANOVA. *P < 0.05, ***P < 0.001, ****P < 0.0001. ns, not significant.

Additionally, flow cytometry was used to assess AMG’s effects on apoptosis in MGO-stimulated HUVECs ( Figure 2A–D). As shown in Figure 2D, MGO increased apoptosis to 14.73% (vs 3.05% in CT). However, it decreased to a normal state in the AMG group (2.65%). ROS fluorescence peaks shifted progressively rightward (AMG < CT < MGO), with MGO showing the highest intensity ( Figure 2E,F), confirming elevated ROS levels in the MGO group. AMG attenuated MGO-induced ROS elevation (134,469 vs 267,605 RFU; RFU: relative fluorescence units), alleviating the rightward peak shift observed in MGO-treated cells. The western blot results showed that under MGO stimulation, endothelial cells exhibited a marked reduction in eNOS and CD31 expression alongside elevated NF-κB levels ( *P*  < 0.05; Figure 2G–K). AMG treatment alleviates MGO-induced damage by upregulating eNOS and CD31 and downregulating NF-κB, demonstrating its significant endothelial protective effects. These benefits may be indirectly mediated by reduced oxidative stress following AGEs diminishment.

### AMG rescues MGO-impaired endothelial glycocalyx structure and function

**Figure 3:**
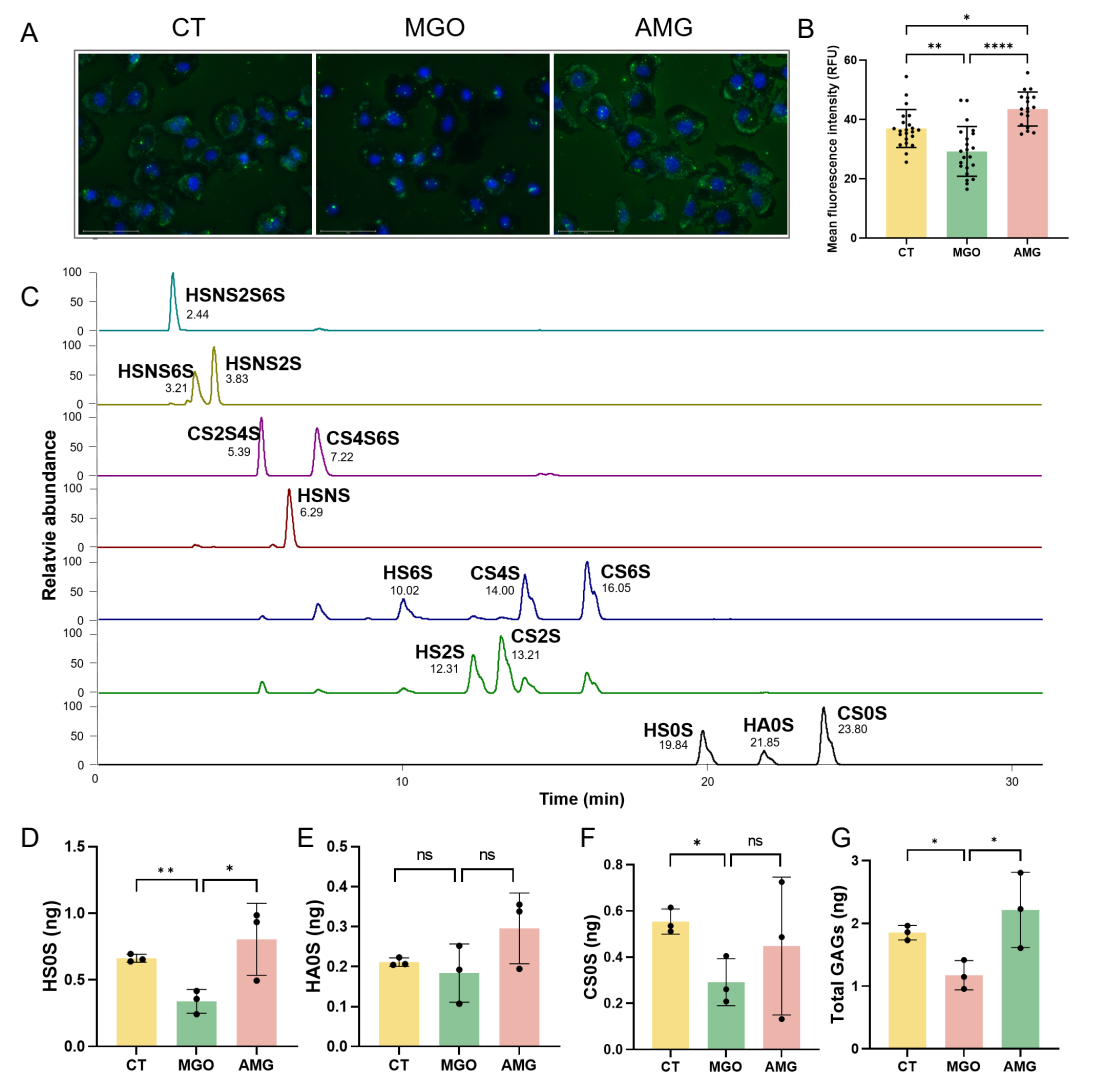
Figure 3 AMG rescues MGO-impaired endothelial glycocalyx structure and function (A) Glycocalyx fluorescence on cell surfaces in CT, MGO, and AMG groups (40× magnification). (B) Statistical analysis of average glycocalyx fluorescence intensity (CT: n = 22, MGO: n = 22, AMG: n = 18). (C) LC-MS/MS MRM analysis of major disaccharide extracted ion chromatograms on endothelial cell surfaces. (D–F) Expression levels of the 3 most abundant disaccharides in CT, MGO, and AMG groups. (G) Statistical analysis of total GAGs content on endothelial cell surfaces after treatment with CT, MGO, and AMG. Data were analyzed by one-way ANOVA and an unpaired Student’s t test. *P < 0.01, **P < 0.01, ****P < 0.0001. ns, not significant.

The GAGs in the endothelial surface form a highly negatively charged glycocalyx. We employed a highly positively charged ScGFP as a probe to label GAGs. Fluorescence imaging of the CT group revealed distinct green fluorescence on the cell membrane, displaying an intact membrane structure. In contrast, the MGO group showed disrupted annular structures of the glycocalyx, with only residual fragmented punctate fluorescence. Notably, AMG intervention restored the original integrity of the glycocalyx ( Figure 3A). Statistical analysis showed that MGO significantly reduced green fluorescence intensity (CT: 37.01 RFU; MGO: 29.27 RFU; *P* = 0.0016), while AMG treatment markedly increased it (AMG: 43.59 RFU; *P*  < 0.0001; Figure 3B).

To further elucidate the compositional changes in glycocalyx GAGs, quantitative analysis of disaccharide units was performed following extraction and enzymatic digestion of cell glycans. Figure 3C displays the extracted ion chromatograms of 14 disaccharides derived from enzymatic digestion of GAGs analyzed by LC-MS/MS MRM. Targeted quantification of disaccharide subunits revealed that HS0S (GlcA-β1-4GlcNAC), HA0S (GlcA-β1-3GlcNAc), and CS0S (GlcA-β1-3GalNAc) were the three most abundant disaccharides ( Figure 3D–F). The structures of other disaccharides are shown in Supplementary Table S1. Statistical analysis revealed significant decreases in HS0S (CT: 0.6620 ng; MGO: 0.3387 ng; *P =* 0.0039) and CS0S (CT: 0.5537 ng; MGO: 0.2913 ng; *P =*  0.0171) expression in the MGO group compared to CT, while HA0S showed a non-significant downward trend (CT: 0.2110 ng; MGO: 0.1837 ng; *P =*  0.5551). The AMG increased all three disaccharides, with HS0S demonstrating a statistically significant elevation (MGO: 0.3387 ng; AMG: 0.8043 ng; *P =* 0.0470). The total GAGs content ( Figure 3G) was also significantly reduced by MGO stimulation (CT: 1.857 ng; MGO: 1.173 ng; *P =*  0.0107), whereas AMG intervention restored it (AMG: 2.215 ng; *P =*  0.0487), further confirming AMG’s protective and reparative effects on endothelial glycocalyx integrity.

### AMG improves the cellular permeability compromised by MGO

Given that AGEs compromise glycocalyx integrity, FITC-BSA fluorescence was employed to assess endothelial permeability changes. Supplementary Figure S2A–C show the fluorescence of cells and their views for each group. In the CT group ( Supplementary Figure S2A), the FITC-BSA was mostly outside the membrane, and a small amount passed through the membrane. The MGO group ( Supplementary Figure S2B) showed shrunken cells with internalized fluorescence, indicating membrane damage with uneven and significantly enhanced fluorescence intensity. In the AMG group ( Supplementary Figure S2C), the cell morphology showed less change compared to the CT group, with fluorescence outside the membrane, resulting in weaker fluorescence intensity. Statistical analysis ( Supplementary Figure S2D) indicated that the positivity rate in the MGO group (83.38%) was significantly higher than that in the CT group (15.87%) and the AMG group (15.45%), suggesting that MGO stimulation leads to cell membrane disruption and increased permeability. The action of AMG antagonizes the destructive effects of MGO, resulting in a similar level of permeability compared to that of the CT group.

### AMG ameliorates metabolic homeostasis compromised by MGO

Through comprehensive metabolomics analysis, 695 distinct metabolites were identified. The principal component analysis (PCA) score plot reveals a distinct clustering pattern, with the MGO group exhibiting a pronounced separation from the AMG and CT groups ( Supplementary Figure S3A). Conversely, the AMG and CT group samples display a more proximate clustering, suggesting a greater degree of metabolic similarity between them.

To identify differentially abundant metabolites, volcano plots were used to perform pairwise comparisons among the CT, MGO, and AMG groups (fold change > 1.3, *P <*  0.05; Supplementary Figure S3B–D). In the MGO/CT comparison, a total of 235 differentially generated metabolites were identified, comprising 105 upregulated and 130 downregulated metabolites ( Supplementary Figure S3B). In the AMG/MGO comparison, 225 differentially generated metabolites were detected, with 121 upregulated and 104 downregulated metabolites ( Supplementary Figure S3C). Finally, in the AMG/CT comparison, 118 differentially generated metabolites were discerned, consisting of 64 upregulated and 54 downregulated metabolites ( Supplementary Figure S3D).

**Figure 4:**
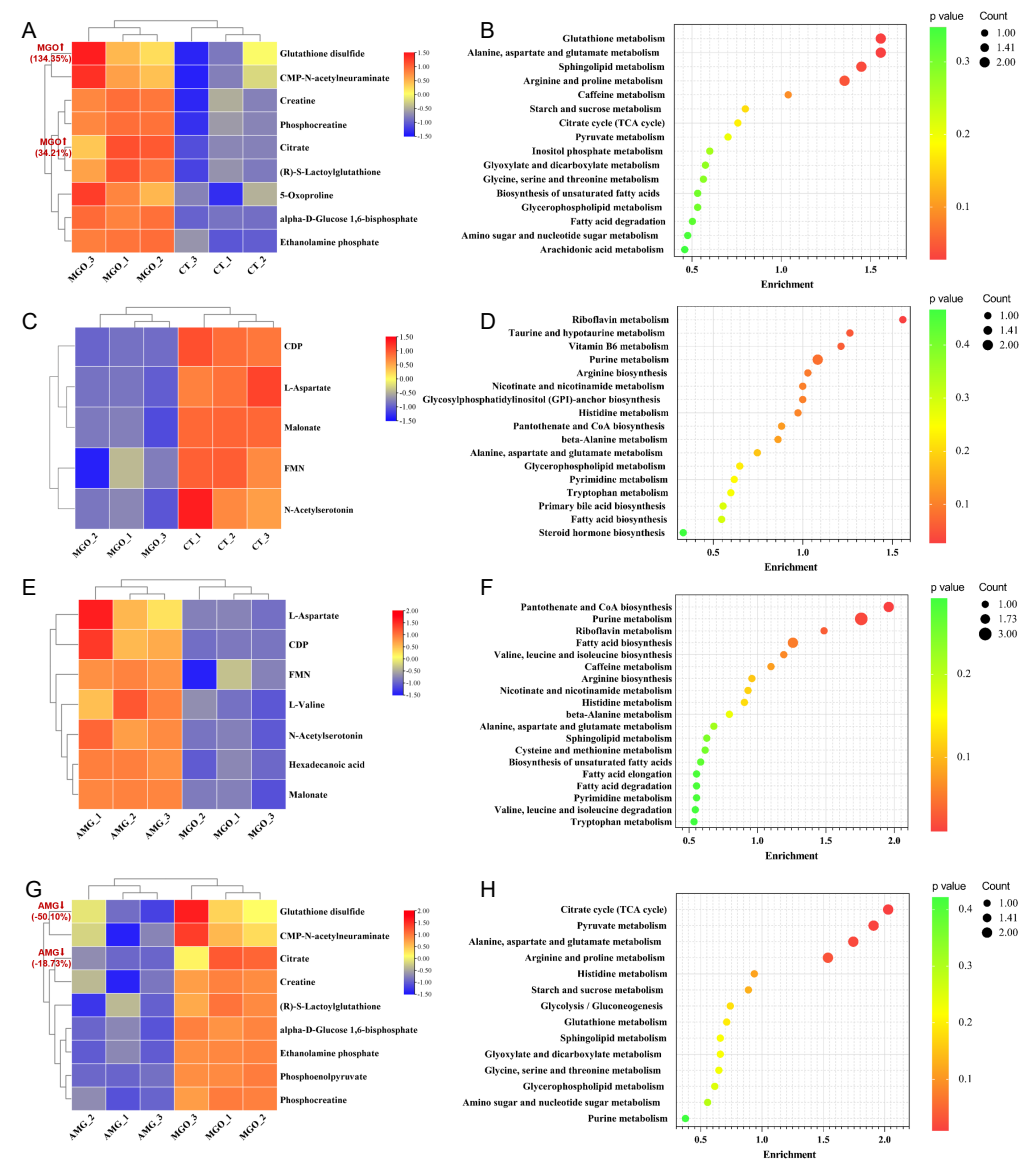
Figure 4 AMG ameliorates metabolic homeostasis compromised by MGO (A) Heatmap of peak areas for upregulated metabolites in MGO/CT. (B) Bubble plot of metabolic pathways enriched by upregulated metabolites in MGO/CT. (C) Heatmap of peak areas for downregulated metabolites in MGO/CT. (D) Bubble plot of metabolic pathways enriched by downregulated metabolites in MGO/CT. (E) Heatmap of peak areas for upregulated metabolites in AMG/MGO. (F) Bubble plot of metabolic pathways enriched by upregulated metabolites in AMG/MGO. (G) Heatmap of peak areas for downregulated metabolites in AMG/MGO. (H) Bubble plot of metabolic pathways enriched by downregulated metabolites in AMG/MGO.

To further elucidate the biological processes involved in upregulated and downregulated metabolites, the KEGG database was selected to enrich metabolite-related pathways. The results showed that 10 changed metabolites [citrate, phosphoenolpyruvate, α-D-glucose 1,6-diphosphate (α-D-G-1,6-P), (R)-S-lactoylglutathione, glutathione disulfide (GSSG), 5-oxoproline (5-OP), creatine, phosphocreatine, ethanolamine phosphate, cytidine monophosphate-N-acetylneuraminic acid (CMP-Neu5Ac)] ( Figure 4A,G) were enriched in 8 pathways, including the citrate cycle (TCA cycle), glyoxylate/dicarboxylate metabolism, starch/sucrose metabolism, pyruvate metabolism, glutathione metabolism, glycine/serine/threonine metabolism, arginine/proline metabolism, and amino sugar/nucleotide sugar metabolism ( Figure 4B,H).

**Figure 5:**
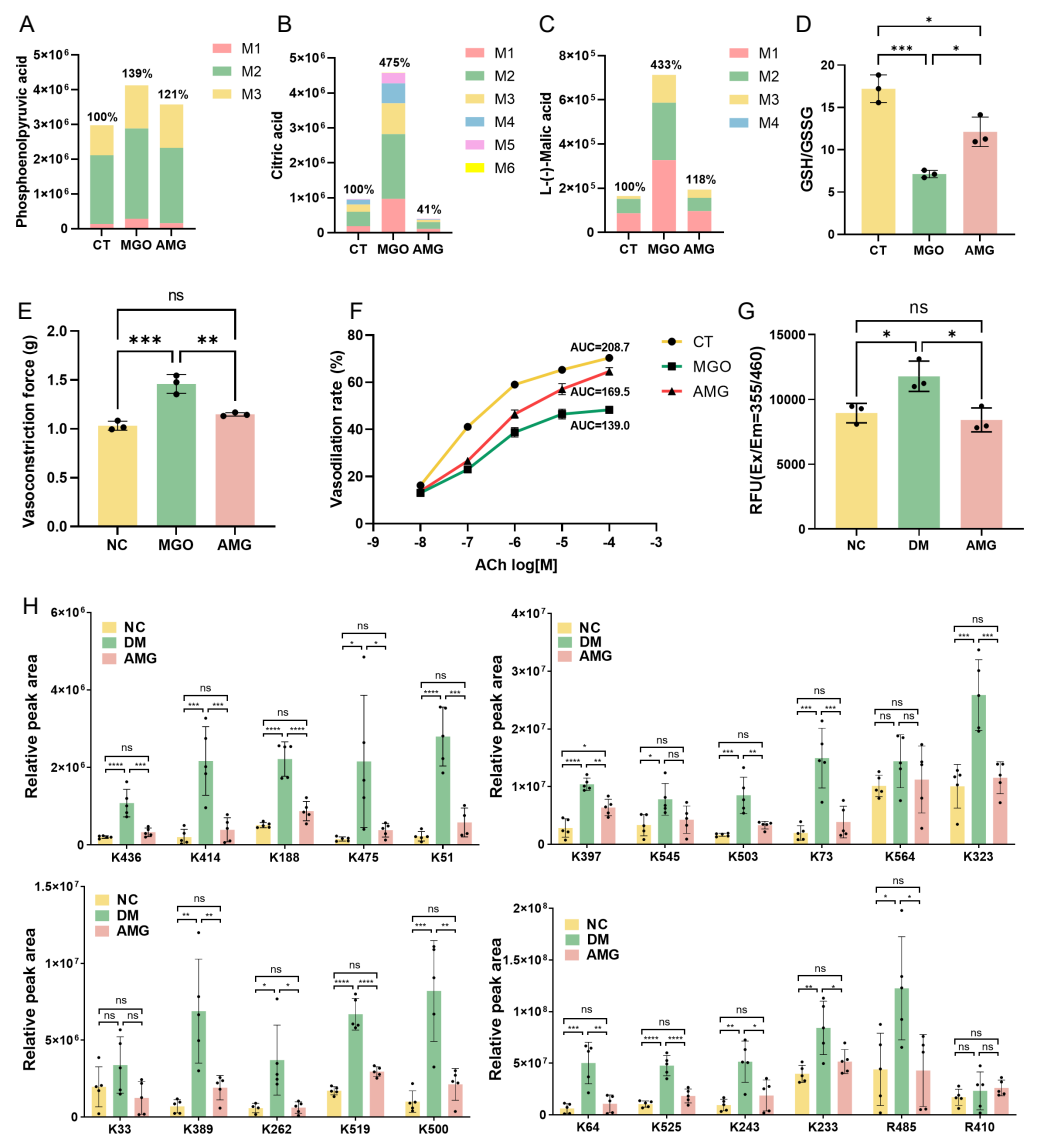
Figure 5 AMG improves metabolic pathways, restores vascular vasoconstriction/vasodilation force, and reduces non-enzymatic glycation sites (A–C) Relative quantification of 13C-labeled phosphoenolpyruvic acid, citric acid, and L-(-)-malic acid in the TCA cycle. Mn represents the molecule with n 13C atom substitutions. (D) Changes in the GSH/GSSG ratio (n = 3). (E) Statistical analysis of vasoconstriction force in each group (n = 3). (F) The trend of changes in endothelial-dependent vasodilation force between each group (n = 3). (G) Plasma AGEs fluorescence levels after drug intervention in mice (n = 3). (H) Relative area integration of nonenzymatic glycation sites of plasma HSA under 3 treatments (n = 5). Data were analyzed by one-way ANOVA. *P < 0.05, **P < 0.01, ***P < 0.001, ****P < 0.0001. ns, not significant.

Analysis of key metabolites revealed that citrate and glutathione increased by 34.21% and 134.35%, respectively, in the MGO group (vs CT). In the AMG group (vs MGO), their levels decreased by 18.73% and 50.10%, respectively. To orthogonally validate the TCA cycle changes, we cultured HUVECs under different treatments with ^1^ ^3^C-labeled glucose as the sole carbon source to analyze metabolic flux. The ^1^ ^3^C-labeled phosphoenolpyruvic acid (PEP), citric acid and L-(-)-malic acid in the MGO group were increased compared with those in the CT group (PEP: increased to 139%; citric acid: increased to 475%; L-(-)-malic acid: increased to 433%), while AMG reduced them (compared with the MGO group; PEP: reduced by 18% to 121%; citric acid: reduced by 434% to 41%; L-(-)-malic acid; reduced by 315% to 118%) ( Figure 5A–C). These results indicate that the overall TCA cycle was accelerated by MGO-induced oxidative stress and that AMG alleviated this state. The changes in ^1^ ^3^C-labeled metabolites were consistent with the results of metabolomics analysis. This confirms the compensatory ATP production of cells in response to oxidative stress and the antagonistic effect of AMG on oxidative stress. Furthermore, targeted metabolomics data of glutathione metabolism indicate that the GSH/GSSG ratio ( Figure 5D) was significantly reduced in the MGO group, while AMG cotreatment effectively alleviated this trend, confirming that AMG mitigates oxidative stress-induced injury.

Seven changed metabolites [L-aspartate (L-Asp), flavin mononucleotide (FMN), L-valine, hexdecanoic acid, malonate, N-acetylserotonin (NAS), and CDP] ( Figure 4C,E) were enriched in 9 metabolic pathways ( Figure 4D,F), including β-alanine metabolism, arginine biosynthesis, nicotinamide/nicotinamide metabolism, alanine/aspartate/glutamate metabolism, riboflavin metabolism, pantothenate/CoA biosynthesis, fatty acid synthesis, tryptophan metabolism and pyrimidine metabolism. The functions of these pathways are mainly focused on scavenging ROS and inhibiting the production of inflammatory factors and the occurrence of glycation in a physiological state. Compared with the MGO group, the AMG/CT group exhibited only minor alterations, as detailed in Supplementary Figure S4.

### AMG attenuates MGO-induced vascular dysfunction and protein glycation *in vivo*

An *ex vivo* aorta ring assay was performed to explore vasoconstriction and endothelial-dependent vasodilation force. Statistical analysis confirmed significantly enhanced vasoconstriction force in MGO (1.4598 g) vs NC (1.0320 g) ( *P*  < 0.001), with partial attenuation by AMG (1.1479 g) ( *P*  < 0.01; Figure 5E). Endothelial-dependent vasodilation was pre-contracted with NE followed by cumulative addition of ACh to stimulate endothelial cells to release NO. The results showed that Ach-induced vasodilation was impaired in the MGO group (AUC = 139.0 vs NC, AUC = 208.7; AUC: area under the curve), with partial restoration in the AMG group (AUC = 169.5 vs MGO) ( Figure 5F). These results, consistent with prior cellular studies, confirm AMG’s protective role in the eNOS/NO pathway.

After 45 days of AMG intervention in the T2DM mice, plasma AGEs were significantly elevated in DM and reduced by AMG treatment (NC: 8948 RFU; DM: 11,789 RFU; AMG: 8420 RFU; *P*  < 0.05; Figure 5G). These results confirm AMG’s inhibition of protein glycation and AGEs formation, consistent with cellular findings. NanoLC-MS/MS was used for qualitative and quantitative analysis of non-enzymatic glycation sites of albumin in mouse serum ( Figure 5H). A total of 22 glycation sites were identified. Among these, 18 sites (K436, K414, K188, K475, K51, K397, K503, K73, K323, K389, K262, K519, K500, K64, K525, K243, K233, and R485) showed significantly increased glycation in diabetic mice, which was reduced by AMG treatment. Notably, sites R485, K233, K64, K243, and K525 showed marked differences. Despite lacking statistical significance, the remaining 4 sites (K545, K564, K33, and R410) exhibited a consistent protective trend with AMG intervention. This confirmed that AMG can protect 81.82% of the basic amino acids on albumin from glycation.

### AMG protects against endothelial pathology and inflammation in diabetic mice

**Figure 6:**
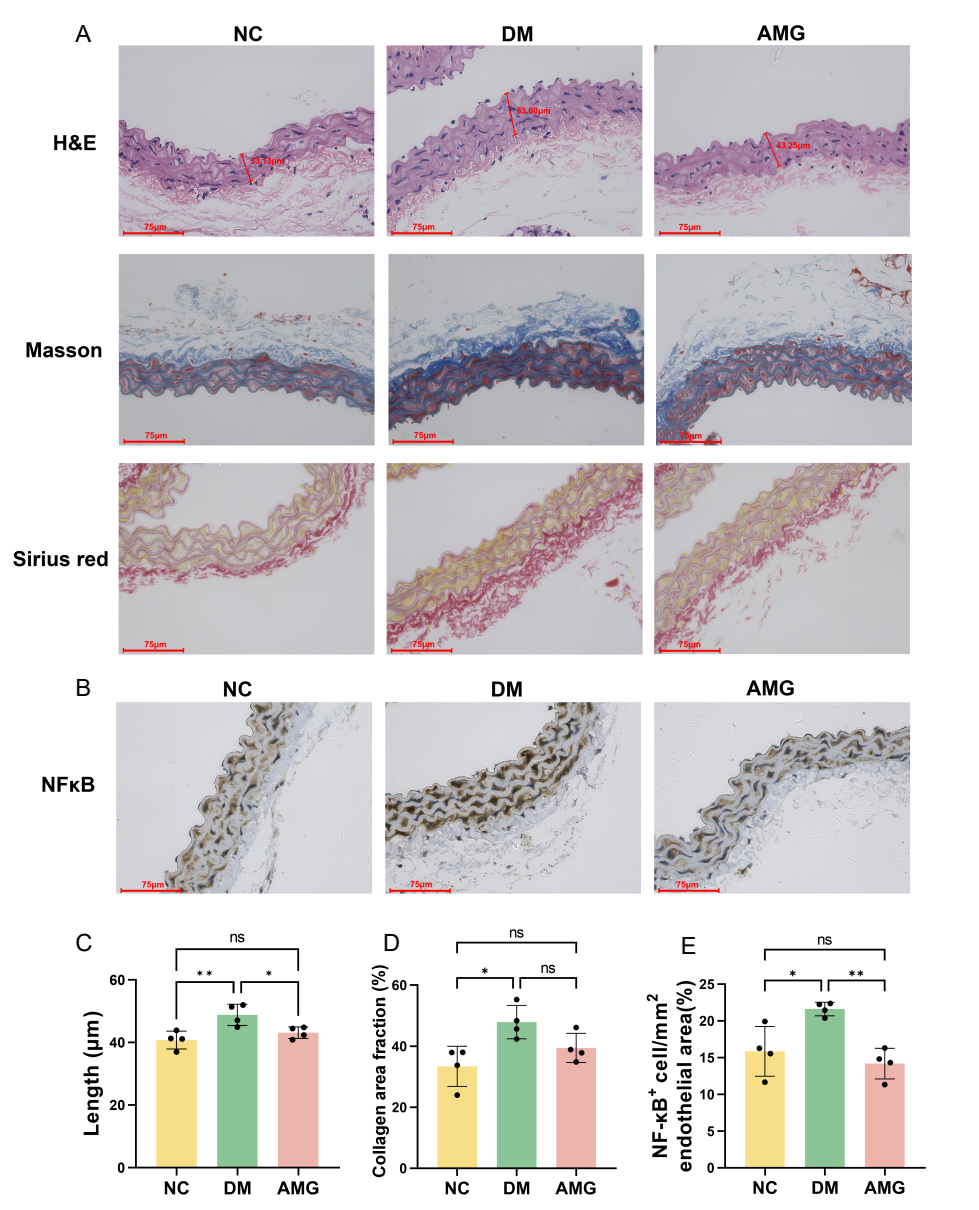
Figure 6 AMG protects against endothelial pathology and inflammatory dysregulation in diabetic mice (A) Thoracic aorta paraffin sections from the NC, DM, and AMG groups stained with H&E, Masson, and Sirius red, respectively. H&E: nuclei appear blue-purple, cytoplasm red. Masson: collagen fibers appear blue, muscle fibers appear purple-red. Sirius red: Collagen fibers appear red with a yellow background, and type I collagen fibers adhere to the outer vascular wall, appearing as orange or bright red thick fibers. (B) Immunohistochemistry of NF-κB in thoracic aortas from the NC, DM, and AMG groups. Paraffin section staining techniques were employed to process the thoracic aortas from mice in the NC, DM, and AMG groups. Sacle bar: 75 μm. (C) Vascular wall length in H&E staining among the three groups of mice (n = 4). (D) Statistical analysis of collagen area fraction in Masson-stained sections (n = 4). (E) Area ratio of positive cells in the NF-κB immunohistochemistry (n = 4). Data were analyzed by one-way ANOVA. *P < 0.05, **P < 0.01. ns, not significant.

The pathological conditions of the vessels were observed under a live cell imaging system ( Figure 6A). H&E staining revealed deeper nuclear staining of endothelial cells, irregular thickening of the arterial intima, and indistinct structural layers of the artery in DM mice. Following AMG intervention, the pathological thickening of the vessels was ameliorated (NC: 40.79 μm; DM: 48.83 μm; AMG: 43.14 μm) ( Figure 6C). Masson’s trichrome staining demonstrated deeper blue staining in the tunica media and tunica adventitia, indicating severe fibrosis in diabetic mice (NC: 33.43%; DM: 47.93%; AMG: 39.44%) ( Figure 6D). Moreover, Sirius red staining confirmed AMG’s suppression of type I collagen deposition and elastic fiber fragmentation, demonstrating its protective effects against diabetic vascular injury.

Furthermore, the immunohistochemical results of the inflammatory factor NF-κB, which is associated with endothelial injury, are shown in Figure 6B. Under DM conditions, the staining intensity of NF-κB was enhanced throughout the endothelial layer of the vessels (NC: 15.85%; DM: 21.62%, *P* = 0.0176). AMG intervention reduced the activation of NF-κB, restoring it to normal levels (14.19%, *P* = 0.0040; Figure 6E).

## Discussion

The damage to vascular endothelial cells caused by chronic hyperglycemia in diabetes is mainly mediated by AGEs produced by non-enzymatic glycation. AGEs accumulation not only disrupts the original cross-links in endothelial collagen, impairing tissue functionality and inducing structural stiffening [16] but also exacerbates vascular dysfunction by engaging RAGE and activating oxidative stress and inflammatory signals. As a carbonyl scavenger, AMG’s free amino group selectively binds to glycation intermediates such as MGO, thereby inhibiting AGEs formation and alleviating endothelial cell inflammatory damage [17].

In the HSA/glucose co-culture system, AMG significantly attenuated AGEs fluorescence intensity, indicating its capacity to inhibit protein glycation under hyperglycemic conditions. Meanwhile, AMG intervention significantly decreased the elevated levels of AGEs caused by MGO in HUVECs culture, revealing its role as an MGO scavenger in endothelial protection. Such protection is evident in improved cell morphology, as well as cell viability, proliferation, apoptosis and ROS levels. MGO induces cells to generate a substantial quantity of ROS, thereby suppressing the eNOS/NO pathway and CD31 expression while upregulating NF-κB, collectively impairing vascular homeostasis. Notably, AMG counteracted the significant suppression of the eNOS/NO pathway induced by both MGO and L-NAME, effectively alleviating these effects. These findings collectively provide preliminary evidence that AMG ameliorates multiple aspects of HUVECs functionality against MGO-induced injury.

The endothelial glycocalyx preserves vascular barrier integrity, regulates vasoconstriction, and resists inflammation, but is vulnerable to degradation under oxidative stress and inflammation [18]. Using fluorescence imaging, disaccharide quantification, and endothelial permeability assays, this study provides evidence that AMG confers endothelial protection by preserving glycocalyx integrity. As an MGO scavenger, AMG may reduce the enzymatic degradation of GAGs through its indirect reduction of oxidative stress, thereby alleviating inflammation and improving the vascular protective function of GAGs under hyperglycemic conditions.

**Figure 7:**
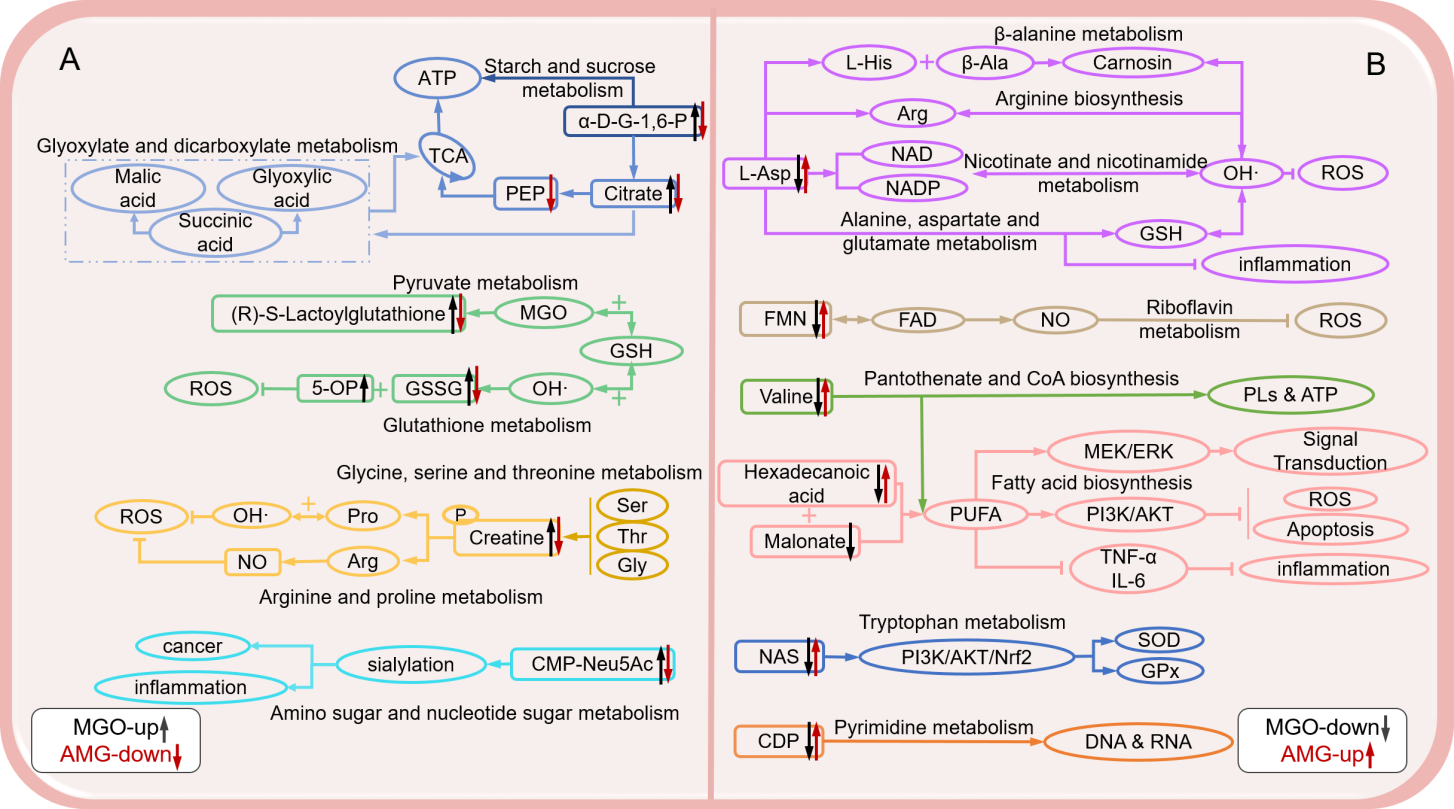
Figure 7 Schematic diagrams of pathways affected by MGO and AMG intervention (A) Metabolites upregulated in MGO and downregulated in AMG and their associated pathways. (B) Metabolites downregulated in MGO and upregulated in AMG and their associated pathways. In pathway diagrams, a flat-headed arrow denotes inhibition, while a sharp-headed arrow indicates promotion.

Metabolomics analysis was conducted to validate the metabolic basis of phenotypic differences in the glycation model. The MGO/CT-upregulated metabolites and the AMG/MGO-downregulated metabolites were enriched in 16 and 14 pathways, respectively, with 8 shared pathways. These pathway alterations reflect MGO-induced excessive endogenous oxidative stress and compensatory ATP production ( Figure 7A).

MGO upregulated citrate, which was enriched in the TCA cycle, glyoxylate/dicarboxylate metabolism and starch/sucrose metabolism. Integrated analysis of the three altered pathways, combined with ^1^ ^3^C-labeled TCA cycle flux validation, revealed compensatory ATP production in cells in response to oxidative stress and the antagonistic effect of AMG on oxidative stress. The upregulation of (R)-S-Lactoylglutathione, which is a direct reaction product of MGO and glutathione (GSH), and its enrichment in pyruvate metabolism collectively demonstrate the GSH-dependent detoxification pathway against MGO-induced oxidative stress [19], while AMG suppressed it by directly trapping MGO, preventing oxidative stress at its source [17].

Notably, the elevated levels of GSSG and 5-OP in the MGO group provide evidence for the enrichment of the glutathione metabolism pathway. As a key antioxidant, GSH neutralizes ROS (generating GSSG, an intracellular oxidative stress biomarker), protecting cells from free radical damage [20]. The decreased GSH/GSSG ratio indicates enhanced cellular oxidative stress under MGO exposure [21]. This oxidative stress was mitigated by AMG intervention, thereby providing solid functional evidence for AMG’s protective role.

Creatine and phosphocreatine were upregulated by MGO but downregulated by AMG. The enrichment of these metabolites in the glycine/serine/threonine and arginine/proline metabolism pathways suggest a coordinated endogenous defense strategy. This strategy potentially involves substrates for creatine synthesis, alongside the anti-AGEs/NO-boosting functions of arginine and the antioxidant/anti-glycation properties of proline [ 22, 23] .

MGO upregulated CMP-Neu5Ac in amino sugar metabolism, potentially enhancing sialylation, a process linked to pro-inflammatory cytokine production in cancers [ 24, 25] . In contrast, AMG reduced sialylation substrate availability, suggesting a mechanism for its anti-inflammatory effects.

On the other hand, metabolites downregulated by MGO (MGO/CT) and upregulated by AMG (AMG/MGO) were enriched in 17 and 19 pathways, respectively, sharing 9 common pathways ( Figure 7B).

The downregulation of L-Asp by MGO was enriched in the β-alanine metabolism, arginine biosynthesis, nicotinamide/nicotinamide metabolism, and alanine/aspartate/glutamate metabolism pathways. L-Asp serves as a key precursor for multiple antioxidant pathways, including carnosine, NAD ^+^, and GSH synthesis, while also reinforcing arginine-mediated antiglycation defense and modulating inflammatory responses via the GSH-dependent redox system [ 26– 29] . AMG upregulates L-Asp, suggesting that it may confer combined benefits of antioxidant, antiglycation, and anti-inflammatory activities.

FMN, the bioactive riboflavin derivative, functions as a critical redox cofactor in energy metabolism [30]. In synergy with flavin adenine dinucleotide (FAD), it potentiates NO synthesis while scavenging ROS [31]. MGO reduces NO and increases ROS, whereas AMG alleviates these effects, demonstrating its endothelial protective role through redox regulation, as validated in our eNOS/NO pathway experiments.

MGO downregulated valine, a metabolic change enriched in the pantothenate/CoA biosynthesis pathway, implying potential disruptions in phospholipid synthesis, fatty acid metabolism, and cellular energy supply [32]. AMG upregulated valine, hexadecanoic acid and malonate to generate polyunsaturated fatty acids (PUFAs). PUFAs may activate the MEK/ERK and PI3K-AKT pathways while suppressing TNF-α and IL-6 production via NF-κB inhibition [ 33, 34] , thereby ultimately disrupting the ROS-inflammation-apoptosis cascade.

Additionally, MGO downregulates NAS in tryptophan metabolism and disturbs PI3K/AKT/Nrf2-mediated cytoprotection (anti-apoptotic/anti-inflammatory/antioxidant/autophagy regulatory) [35]. The upregulation of NAS by AMG may protect endothelial defense mechanisms against free radical damage.

Finally, the enrichment of CDP, a central metabolite in the pyrimidine metabolism pathway essential for DNA/RNA synthesis [36], implies that AMG may alleviate MGO-induced deficits in cell proliferation by restoring pyrimidine metabolism.

*In vivo* mouse studies confirmed that MGO impairs endothelial function by attenuating eNOS-derived NO production and diminishing ACh-induced vasodilation while increasing vasoconstriction. AMG treatment effectively restored normal eNOS/NO pathway activity and vascular responsiveness, demonstrating its protective role in maintaining endothelial function through NO-mediated mechanisms, consistent with cellular and molecular findings.

Beyond eNOS/NO pathway-mediated protection, AMG’s effects on diabetic glycation were investigated through plasma AGEs quantification and vascular histopathology. Diabetic mice exhibited elevated plasma AGEs levels compared to the NC and AMG groups, with NanoLC-MS/MS analysis confirming increased glycation at 22 albumin residues. AMG decreased residue glycation by 81.82% by trapping reactive dicarbonyls ( *e*. *g*., MGO, glyoxal, glucosone) and suppressing AGEs formation. This carbonyl-quenching effect preserves albumin integrity and mitigates AGEs-driven endothelial damage in diabetes.

Histopathological and immunohistochemical analyses revealed that diabetic mice developed significant vascular damage, including basement membrane thickening, collagen disorganization, and elevated NF-κB expression in the endothelium. AMG treatment effectively mitigated these pathological changes by reducing AGEs-mediated collagen cross-linking, suppressing AGEs-RAGE signaling, and restoring vascular structural integrity [37].

In contrast to metformin’s AMPK-dependent improvement of insulin sensitivity [38], AMG confers vascular protection through direct inhibition of AGEs formation and preservation of the endothelial glycocalyx. For future research on the synergistic protective effects of AMG and metformin, it is hypothesized that a synergistic effect may exist between metformin’s systemic metabolic control and AMG’s targeted anti-AGEs action, offering superior vascular protection compared to either monotherapy.

The main limitations include the correlative nature of the metabolomics data, which requires functional validation to confirm causal pathway mechanisms, and a study design that did not evaluate sex-specific effects and warrants larger cohort validation due to the sample size. Additionally, the high MGO concentration may induce excessive glycation damage, and the lack of pharmacokinetic/pharmacodynamic (PK/PD) data for AMG hinders dose optimization. Furthermore, the causal role of the eNOS/NO pathway identified in cellular experiments requires further validation using *eNOS*-knockout animal models.

In summary, this study demonstrates AMG’s multifaceted protection against diabetic endothelial injury by trapping glycation intermediates to inhibit AGEs formation, restoring glycocalyx integrity and the eNOS/NO pathway, and exerting a combined antioxidant/antiglycation/anti-inflammatory profile via metabolic regulation effects. Animal studies have confirmed that AMG reduces protein glycation and improves vascular function, supporting its targeted strategies for diabetic vascular complications.

## Supporting information

25763Supplementary_Data

## Supplementary Data

Supplementary data are available at *Acta Biochimica et Biophysical Sinica* online.
