# Supplementary material for: Multifaceted elucidation of aminoguanidine in protecting against diabetes-induced vascular endothelial injury: Aminoguanidine protects against diabetic endothelial injury
Source: Acta Biochim Biophys Sin (Shanghai). 2026 Feb 3;58(7):1519–32. doi: 10.3724/abbs.2026005 (PMC13448999; doi:10.3724/abbs.2026005)
Supplement: Supplementary file 1 [file 25763Supplementary_Data.docx]

**Supplementary Figure S1. AMG ameliorates MGO-induced cellular morphological change** Cellular morphology changes in CT, MGO, and AMG groups (20× magnification).

**Supplementary Figure S2. AMG improves the cellular permeability compromised by MGO** (A) Fluorescence intensity of cell permeability in CT group. (B) Fluorescence intensity of cell permeability in MGO group. (C) Fluorescence intensity of cell permeability in AMG group. (D) Statistical analysis of cell permeability positivity rate in three groups (*n* = 3). Data were analyzed by one-way ANOVA; *P <* 0.05 indicates significance.

**Supplementary Figure S3. Differential metabolite regulation by AMG versus MGO intervention** (A) PCA plot of 695 metabolites identified by LC-MS/MS in CT, MGO, AMG, and QC groups. (B) Volcano plot of MGO/CT comparison. (C) Volcano plot of AMG/MGO comparison. (D) Volcano plot of AMG/CT comparison. Red indicates upregulated and blue indicates downregulated metabolites.

**Supplementary Figure S4.** **AMG regulates MGO-induced metabolic imbalance** (A) Heatmap of peak areas for upregulated metabolites in AMG/CT. (B) Bubble plot of metabolic pathways enriched by upregulated metabolites in AMG/CT. (C) Heatmap of peak areas for downregulated metabolites in AMG/CT. (D) Bubble plot of metabolic pathways enriched by downregulated metabolites in AMG/CT.

**Supplementary Table S1. Structure of disaccharide units of GAGs in endothelial glycocalyx**

| **Disaccharide** | **Structure** |
| --- | --- |
| HSNS2S6S | GlcA2S-β1-4GlcNS6S |
| HSNS6S | GlcA-β1-4GlcNS6S |
| HSNS2S | GlcA2S-β1-4GlcNS |
| CS2S4S | GlcA2S-β1-3GalNAc4S |
| CS4S6S | GlcA-β1-3GalNAc4S6S |
| HSNS | GlcA-β1-4GlcNS |
| HS6S | GlcA-β1-4GlcNAc6S |
| CS4S | GlcA-β1-3GalNAc4S |
| CS6S | GlcA-β1-3GalNAc6S |
| HS2S | GlcA2S-β1-4GlcNAc |
| CS2S | GlcA2S-β1-3GalNAc |
| HS0S | GlcA-β1-4GlcNAc |
| HA0S | GlcA-β1-3GlcNAc |
| CS0S | GlcA-β1-3GalNAc |

GAGs: glycosaminoglycans; HS: heparan sulfate; CS: chondroitin sulfate; HA: hyaluronic acid; GlcA: glucuronic acid; GalNAC: N-acetylgalactosamine; GlcNAC: N-acetylglucosamine; GlcNS: N-sulfoglucosamine.
